# Temporo-spatial distribution of stem cell markers CD146 and p75NTR during odontogenesis in mice

**DOI:** 10.1590/1678-7757-2021-0138

**Published:** 2021-09-20

**Authors:** Aline QUEIROZ, Cibele PELISSARI, Victor Elias ARANA-CHAVEZ, Marília TRIERVEILER

**Affiliations:** 1 Universidade de São Paulo Faculdade de Odontologia Departamento de Estomatologia São PauloSP Brasil Universidade de São Paulo, Faculdade de Odontologia, Departamento de Estomatologia, Disciplina de Patologia Oral e Maxilofacial, Laboratório de Biologia de Células-Tronco em Odontologia LABITRON, São Paulo, SP, Brasil.; 2 Universidade de São Paulo Faculdade de Odontologia Departamento de Biomateriais e Biologia Oral São PauloSP Brasil Universidade de São Paulo, Faculdade de Odontologia, Departamento de Biomateriais e Biologia Oral, São Paulo, SP, Brasil.

**Keywords:** Tooth development, Odontogenic stem cells, Odontogenesis, p75 neurotrophin receptor, CD146 antigen

## Abstract

Mesenchymal and epithelial stem cells were identified in dental tissues; however, knowledge about the odontogenic stem cells is limited, and there are some questions regarding their temporo-spatial dynamics in tooth development.

**Objective:**

Our study aimed to analyze the expression of the stem cell markers CD146 and p75NTR during the different stages of odontogenesis.

**Methodology:**

The groups consisted of 13.5, 15.5, 17.5 days old embryos, and 14 days postnatal BALB/c mice. The expression of CD146 and p75NTR was evaluated by immunohistochemistry.

**Results:**

Our results showed that positive cells for both markers were present in all stages of tooth development, and the number of positive cells increased with the progression of this process. Cells of epithelial and ectomesenchymal origin were positive for CD146, and the expression of p75NTR was mainly detected in the dental papilla and dental follicle. In the postnatal group, dental pulp cells were positive for CD146, and the reduced enamel epithelium and the oral mucosa epithelium showed immunostaining for p75NTR.

**Conclusions:**

These results suggest that the staining pattern of CD146 and p75NTR underwent temporal and spatial changes during odontogenesis and both markers were expressed by epithelial and mesenchymal cell types, which is relevant due to the significance of the epithelial-ectomesenchymal interactions in tooth development.

## Introduction

Odontogenesis in mammals begins with the migration of neural crest cells derived from the ectoderm. These cells acquire mesenchymal characteristics and are considered ectomesenchymal cells. The epithelial cells proliferate and migrate into the ectomesenchyme, and interactions between these two tissues form a continuous, thickened, so-called primary epithelial band. Dental lamina differentiates from the primary epithelial band and invaginates into the underlying ectomesenchyme to form the epithelial buds. Then, the dental germs undergo subsequent histological stages of tooth development: bud, cap, bell, crown, and root.^1^

Epithelial-ectomesenchymal interactions essential for the development of all dental tissues involve a series of molecular and signaling events,^2^ in which stem cells play a central role, especially the embryonic ones.^3^ Sources of adult mesenchymal stem cells (MSCs) were already obtained from different dental tissues;^4^ several studies demonstrated their promising use for tooth repair and regeneration and for cell-based therapies in regenerative medicine.^3^ On the other hand, the isolation and characterization of stem cells involved in tooth development, i.e., odontogenic stem cells, remains a challenge.

The odontogenic epithelial stem cells (OEpSCs) are present in the dental lamina, epithelial cell rests of Malassez, reduced enamel epithelium, and junctional epithelium.^5^ At the apical end of the murine incisors, there is an epithelial stem cell niche with cells that migrate and differentiate into ameloblasts.^6^ Moreover, ectomesenchymal stem cells (EMSCs) are present in the dental papilla and in the dental follicle that surrounds the tooth germ. These cells are essential to both the epithelium-ectomesenchymal interaction and the tooth morphogenesis.^7,8^

Mesenchymal and epithelial stem cell are identified by using cell surface markers;^9,10^ however, the odontogenic stem cell markers are still not well established. CD146 (MCAM/MUC18) is a transmembrane glycoprotein expressed mainly at the endothelial intercellular junction. It is involved in angiogenesis and may play a role in cell signaling, migration, and differentiation.^11^ This adhesion molecule was initially used as a selective melanoma biomarker,^11,12^ but, currently, it is considered an important marker of MSCs and pericytes.^9,13^ In different tissues such as dental pulp, periodontal ligament and apical papilla, the CD146^+^/STRO-1^+^ profile proved to be the most efficient to isolate MSCs.^14-16^ One of the first studies that identified the perivascular niche of dental pulp stem cells (DPSCs) used CD146 *in situ* expression to locate these cells.^17^ In addition, this surface marker selects cells that are more clonogenic and that show better multi-differentiation properties; it can also separate DPSCs from dental pulp fibroblasts.^18^

The p75 neurotrophin receptor (p75NTR), also known as NGFR/CD271, is a component of the tumor necrosis factor receptor superfamily that commonly binds to neurotrophins, such as neurotrophin-3 and -4/5, nerve growth factor, and brain-derived neurotrophic factor.^19^ p75NTR mediates cellular events such as cell apoptosis, adhesion, differentiation, and invasion, and may be involved in intracellular signaling.^19,20^ Besides being a tumor marker, especially in the gastrointestinal tract,^21^ p75NTR is regarded as a marker of mesenchymal and epithelial stem cells. In populations of embryonic stem cells, p75NTR is a key marker for purifying neural crest cells.^22^ In the pulp of permanent teeth, p75NTR is expressed in a small subpopulation of undifferentiated neural stem cells, which coexpress CD146.^20^ This population has been referred to as ectomesenchymal stem cells.^23,24^ The presence of p75NTR-positive cells in the dental pulp and in the periodontal ligament indicates that neural crest stem cells, with potential for neurodifferentiation, are present in adult tissues.^25^

There are still many questions regarding stem cells and tooth development. The temporo-spatial dynamics among the odontogenic stem cells is unknown, and it is unclear if they proportionally decrease in number or remain constant in the different tissues during development. The murine models give important contributions to the study of cellular and molecular events in odontogenesis; they are relevant for reproducing these mechanisms *in vitro*,^26^ and also for the research in stem cell-based dental regenerative therapies and tooth bioengineering. Therefore, this study aimed to evaluate the immunoexpression of CD146 and p75NTR during the different stages of molar tooth development in mice.

## Methodology

### Animals and tissue preparation

This study was approved by the Ethics Committee on Animal Research of our Institution.

Four male and four female BALB/c mice (*Mus musculus*; 4-week-old; initial body weight: 18-21 g) were housed in individual cages with free access to food and water. They were maintained in a room under a 12-hour light/dark cycle, with a controlled temperature of 23ºC and were allowed to acclimate for 7 days before any procedure.

The four couples were mated overnight, and the day after the mating was designated as “0.5” day of pregnancy if the vaginal plug was present. After mating, the females were individually housed in cages with the same aforementioned conditions, and males were euthanized.

For this qualitative study, each experimental group was composed by the litter of a female. The groups consisted of embryos of 13.5 (E13.5, n=7), 15.5 (E15.5, n=9) and 17.5 (E17.5, n=9) days old, and 14 days postnatal mice (PN14, n=5). In total, 21 embryos (seven mice in each group: E13.5, E15.5 and E17.5) were used to analyze the stages of odontogenesis. In the PN14 group, tooth germs from 5 lower jaws and 5 upper jaws were evaluated.

The mice were euthanized with an intramuscular injection of xylazine (100 mg/mL; Anasedan, Ceva, Paulínia – Brazil) and ketamine (50 mg/mL; Dopalen, Ceva, Paulínia – Brazil). After the euthanasia, the heads of the embryos were collected and processed, whereas, in the postnatal group, the upper and lower jaws were first dissected and then processed. All samples were fixed in 4% formaldehyde for 24 hours at room temperature. The molar regions from E17.5 and PN14 were decalcified in 4.13% EDTA for a period sufficient to achieve complete demineralization. Subsequently, the specimens were dehydrated in ascending concentrations of ethanol, diaphanized and embedded in paraffin.

### Morphological evaluation and immunohistochemistry

For each specimen that composed the sample of this study, 1 representative hematoxylin and eosin histological section was selected, in which at least 1 tooth germ of the corresponding odontogenesis stage could be visualized. Afterwards, serial sections were obtained for immunohistochemistry.

Five-μm thick sections were stained with hematoxylin and eosin and examined in a light microscope for morphological analysis.

For the immunohistochemical study, 3 μm thick sections were dewaxed, rehydrated and pretreated for antigen retrieval (citrate buffer, pH 6.0) for 30 min at 95°C in water bath. Then, the slides were incubated for 30 min in a 6% hydrogen peroxide/methanol (1:1) solution to quench endogenous peroxidase activity. After a water rinse, sections were incubated in Tris-buffered saline (TBS, pH 7.4) for 15 min. Primary monoclonal antibodies against CD146 (clone EPR3208, dilution 1:100, Abcam, Cambridge, UK) and p75NTR (clone D4B3, dilution 1:100, Cell Signaling Technology, Danvers, MA) were incubated for 18 h at 4°C. EnVision+ Dual Link System-HRP (Dako, Carpinteria, CA, USA) was used for 30 min at room temperature to detect the reactions, and diaminobenzidine (DAB+, Dako) was used as the chromogen. Samples were counterstained with Mayer’s hematoxylin and analyzed under a light microscope. All reactions were performed with appropriate positive and negative controls. Negative controls were incubated in the absence of the primary antibody. Oral mucosa sections of human specimens and adult mice were used as positive controls.

The analysis of immunohistochemical reactions was semiquantitative. In every section examined, cells were considered positive or negative for CD146 and p75NTR. The intensity of the immunostaining was not analyzed due to its subjectivity, and the scoring for the expression of both markers was considered: negative (-); up to 5% positive cells (+); from 5% to 50% of positive cells (++); more than 50% positive cells (+++).

## Results

**Figure 1:**
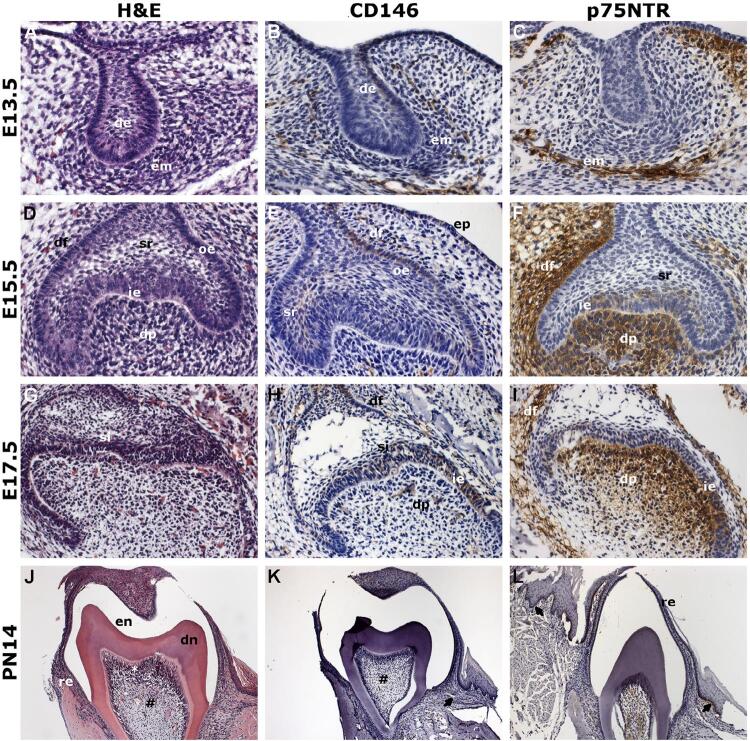
Morphological aspects and CD146 and p75NTR immunoexpression in mice odontogenesis. Rows represent, from top to bottom, the bud (A–C), cap (D–F) and bell (G–I) stages of the tooth germ, and the 14 days postnatal tooth (J–L). (A, D, G, J) show the main morphological characteristics in the different stages. At the bud stage, the immunohistochemical expression of CD146 was detected in the dental epithelium and ectomesenchymal condensation (B). For p75NTR, the immunostaining was restricted to the ectomesenchymal cells (C). At the cap stage, CD146 was found in the outer enamel epithelium, stellate reticulum, dental follicle, and oral epithelium (E). An intense expression of p75NTR was seen in the dental papilla and dental follicle (F). During the bell stage, the CD146 was positive in the inner enamel epithelium, stratum intermedium, dental follicle, and dental papilla (H), and p75NTR was detected in the inner enamel epithelium, dental follicle and dental papilla (I). In the postnatal group, only the dental pulp cells were positive for CD146 (K), while p75NTR immunostaining was seen in the reduced enamel epithelium and oral mucosa epithelium (L). Magnification: A–I, 400× in the original; H–J, 100×

At the bud stage (E13.5), the dental lamina and the oral epithelium were considered as “dental epithelium” due to the difficulty in differentiating them anatomically. We observed that the ectomesenchymal cells gathered around the invaginated dental epithelium. Positive cells for CD146 were detected on the dental epithelium and ectomesenchyme, whereas p75NTR was expressed only in the ectomesenchymal condensation (Figure 1A–C).

(#) dental pulp; (*) odontoblasts; (arrow) oral mucosa epithelium; (de) dental epithelium; (df) dental follicle; (dn) dentine; (dp) dental papilla; (em) ectomesenchymal condensation; (en) enamel space after decalcification; (ep) oral epithelium; (H&E) hematoxylin and eosin; (ie) inner enamel epithelium; (oe) outer enamel epithelium; (re) reduced enamel epithelium; (si), stratum intermedium; (sr) stellate reticulum

During the cap stage (E15.5), as the tooth germ grew, the epithelial bud acquired a cap appearance as well as a higher density of ectomesenchymal cells. From this stage on, the enamel organ and the dental papilla were also observed. CD146-positive cells were detected in the oral epithelium, dental lamina, outer enamel epithelium, dental follicle, and dental papilla. The cells of the inner enamel epithelium and stellate reticulum showed weaker staining for CD146. For p75NTR, the dental follicle, dental papilla, and inner enamel epithelium exhibited positive cells. Cells of the stellate reticulum showed a faint immunoreactivity, whereas the staining in oral epithelium, dental lamina, and outer enamel epithelium was absent (Figure 1D–F).

In the bell stage (E17.5), cells undergo differentiation; the tooth germ shows a pronounced concavity and the presence of the stratum intermedium and the cervical loop. CD146 immunostaining was seen in oral epithelium, dental lamina, dental follicle, dental papilla, and in all enamel organ cells. p75NTR-positive cells were detected in the inner enamel epithelium, stratum intermedium, dental follicle, and dental papilla (Figure 1G–I).

In the PN14 group, the alveolar bone resorption was seen around the germs, and the tooth root and crown were formed. Thus, we observed the enamel space because of the decalcification process, besides the dentine deposition and the dental pulp showing blood vessels. CD146 staining was restricted to dental pulp cells, whereas p75NTR-positive cells were detected in the reduced enamel epithelium and in the oral mucosa epithelium, in the basal layer (Figure 1J–L). Table 1 shows the results of the immunohistochemical analysis.

**Table 1:** Expression of p75NTR and CD146 during tooth development

Group	Localization	p75NTR	CD146
E13.5	Dental epithelium	-	++
	Ectomesenchymal condensation	++	++
E15.5	Oral epithelium	-	+++
	Dental lamina	-	++
	Outer enamel epithelium	-	++
	Inner enamel epithelium	++	+
	Stellate reticulum	+	+
	Dental papilla	+++	++
	Dental follicle	+++	++
E17.5	Oral epithelium	-	+++
	Dental lamina	-	+++
	Outer enamel epithelium	-	++
	Inner enamel epithelium	++	+++
	Stratum intermedium	+	+++
	Stellate reticulum	-	++
	Dental papilla	+++	++
	Dental follicle	+++	++
PN14	Dental pulp cells	-	+
	Odontoblasts	-	-
	Reduced enamel epithelium	++	-
	Basal layer of the oral epithelium	++	-

Negative (-); up to 5% of positive cells (+); from 5% to 50% of positive cells (++); more than 50% of positive cells (+++).

## Discussion

In our study, we showed that all the stages of odontogenesis expressed cells positive for p75NTR and CD146, thus showing the participation of stem cells during tooth initiation, proliferation, morphodifferentiation, and histodifferentiation. Different odontogenic cell populations expressed these markers, which were present since the early stages of tooth development and showed variations in the staining pattern and intensity. Although cell proliferation and mitotic activity are higher in the bud and cap stages,^1^ CD146 and p75NTR-positive cells increased as odontogenesis progressed, with an emphasis on the bell stage.

Besides the regulation of cell death and survival, p75NTR seems to be involved in the differentiation of odontoblasts and ameloblasts.^27,28^ In the primary culture of EMSCs, the p75NTR positive cells showed a greater potential to differentiate into odontoblastic-like cells when compared with those that were negative for the marker.^29^ Also, p75NTR mediated the differential mineralization in rodent EMSCs^7^ and has recently been suggested as a possible regulator of dental morphogenesis.^30^

During the early stages of tooth development, at first, only the ectomesenchymal condensation exhibited cells positive for p75NTR. Then, from the cap stage onwards, both epithelial and mesenchymal cells showed a positive immunolabeling. This pattern was previously described in human fetal teeth, in which positive cells were initially restricted to the ectomesenchyme and then distributed into the undifferentiated cells of the inner enamel epithelium, dental follicle, and nerve fibers.^31^ In the postnatal stages of odontogenesis, only the oral mucosa epithelium showed cells positive for p75NTR.

*In vitro* studies showed that p75NTR is expressed in cultured human dental pulp cells,^20,32^ but, in our sample, the dental pulp was negative for this marker. This result was previously seen in healthy adult human teeth, in which the p75NTR expression was absent in the dental pulp fibroblasts, but positive in nerve fibers.^28^

Neural crest-derived cells and epithelial cells derived from the oral ectoderm are the two populations essential for tooth development. They form, respectively, dental mesenchymal tissues and dental enamel.^3^ In addition, a third population is involved in this process, the glial-derived cells. They are capable of originating dental MSCs and are positive for S-100 protein, Sox10, and p75NTR.^33,34^ Our results showed a restricted expression of p75NTR in epithelial cells and a more widespread expression in the ectomesenchymal derived cells. The immunostaining was particularly intense in the dental papilla and dental follicle as odontogenesis progressed. Thus, we speculate that, in tooth development, p75NTR might be a central marker of cells derived from the neural crest and cells with a glial phenotype, such as Schwann cells, but this requires further investigation.

CD146 is an MSC marker expressed in several tissues, including part of those of dental origin. This marker is also present in the pericytes, which are perivascular cells that surround the endothelium of capillaries and microvessels.^13,35^ CD146 expression was related to the epithelial-mesenchymal transition in cancer;^36,37^ however, to the best our knowledge, this marker has not been reported in epithelial-mesenchymal interaction in tooth development. Although CD146 is a surface molecule used to identify mesenchymal cells, at the embryonic stages of odontogenesis, we observed all layers of the enamel organ immunostaining for CD146. This expression by both epithelial and mesenchymal cell types is interesting because the communication between them is an essential process in odontogenesis.^2^

In the postnatal group, scattered dental pulp cells expressed CD146 while the odontoblasts were negative. A previous study showed that the cells around the pulp blood vessels are CD146-positive in deciduous teeth.^38^ Keller, et al.^39^ (2012) observed an important heterogeneity in the immunostaining of dental pulp cells in development. The blood vessels and pericytes in the fully formed tooth pulp were positive for CD146, while, in the cap stage of odontogenesis, CD146-positive cells were present in the odontogenic epithelium. Further, Xiao, et al.^40^(2017) showed that the cells around odontoblasts, in the cell-rich zone of the pulp of permanent teeth, exhibited a positive staining for CD146.^40^ In our sample, these cells were negative for the marker. We speculate that both the odontoblast layer and the subodontoblastic region may be positive for CD146 in later stages, when the dental pulp is fully developed.

**Figure 2:**
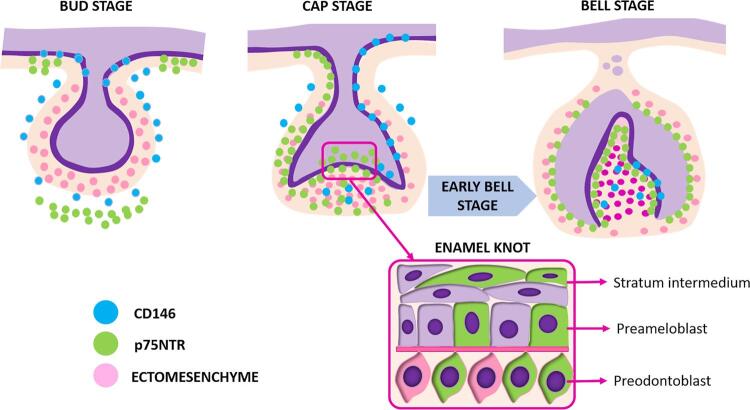
Schematic representation of the immunopositivity for CD146 and p75NTR in the stages of odontogenesis. In the box, we represent an enamel knot during the early bell stage. These signaling centers are responsible for the epithelial-mesenchymal interaction necessary for the regulation of tooth morphogenesis. In this region, both epithelial and ectomesenchymal cells were positive for p75NTR

Figure 2 summarizes our findings with a schematic representation of the relationship between the markers in the stages of odontogenesis. In the enamel knot signaling centers, which participate in regulating tooth morphogenesis, p75NTR was expressed by both epithelial and ectomesenchymal cells, whereas CD146 was absent. This absence of cells positive for CD146 in these zones of the tooth germ may indicate its association with more undifferentiated states of the odontogenic epithelium. These findings may be useful in selecting cell populations for stem cell-based therapies for tooth repair and regeneration.

## Conclusions

In conclusion, the four stages of odontogenesis in mice exhibited positive cells for the stem cell markers CD146 and p75NTR. Furthermore, the staining patterns of both markers underwent temporal and spatial changes during tooth development. In general, the number of positive cells increased with the progression of odontogenesis. Our study demonstrated, for the first time, the expression of the CD146 marker in this process and confirmed the immunostaining for p75NTR in odontogenic and neural crest-derived cells. In addition, both markers were expressed by cells of epithelial and mesenchymal origin, which is interesting due to the crucial epithelial-mesenchymal interactions during odontogenesis.
